# Assessing suicide risk in patients with heart failure: a systematic review and meta-analysis

**DOI:** 10.3389/fpsyt.2025.1674302

**Published:** 2025-09-17

**Authors:** Yujia Zeng

**Affiliations:** Beijing Anzhen Hospital Affiliated to Capital Medical University, Beijing, China

**Keywords:** heart failure, suicide, psychiatry, self-injury, meta-analysis

## Abstract

**Background:**

Heart failure (HF) is a long-lasting and challenging condition. It often relates to issues with mental health and suicidal behavior. However, the exact level of suicide risk in HF patients is not well understood. This systematic review and meta-analysis aimed to assess the connection between HF and suicide risk in adults.

**Methods:**

PubMed, Scopus, and Web of Science were searched up to June 2025. The emphasis was on research that presented outcomes related to suicide in patients with heart failure. Included studies featured adults who were 18 years of age or older and reporting quantitative information, like odds ratios, about suicidal ideation, attempts, or completions. To explore heterogeneity, subgroup analyses were performed based on diagnostic criteria for suicidal behaviors (ICD-9 versus ICD-10) and study design.

**Results:**

Out of 1,643 records, 8 studies were eligible based on the criteria described. The pooled analysis showed a significantly higher risk of suicide in HF patients compared to the general population with no major cardiovascular diseases (OR = 1.62, 95% CI: 1.49-1.74) compared to healthy subjects, with substantial variability (I² = 88.23%). Subgroup analyses revealed that studies using ICD-9 criteria (OR = 1.75, 95% CI: 1.65–1.85) and case-control designs (OR = 1.75, 95% CI: 1.66–1.83) had significantly higher pooled suicide risk estimates than studies using ICD-10 criteria (OR = 1.46, 95% CI: 1.38–1.54) and cohort designs (OR = 1.46, 95% CI: 1.38–1.54). Furthermore, between-group differences were statistically significant (Q = 20.05 and 23.49, p < 0.001), suggesting that diagnostic criteria and study design were significant sources of heterogeneity.

**Conclusion:**

HF is connected to a significantly higher risk of suicide. These results emphasize the importance of regular mental health check-ups and early support in HF care, especially shortly after diagnosis.

## Introduction

Suicide has become a major public health issue worldwide, affecting morbidity and mortality; however, there is less interest and literature on suicide risk in patients with chronic physical conditions (1). Heart failure (HF) is a progressive syndrome affecting millions of people worldwide. It poses serious and disabling physical and psychological challenges that may increase distress, depression, and thoughts of suicide (2, 3). Though it was the subject of over 746,000 deaths worldwide, with nearly 70% men, in the year under consideration by the Global Burden of Disease (GBD) Study 2021, the age-standardized suicide mortality rate has been generally on the decline since 1990; very high in some regions, with much of those being in Eastern Europe and sub-Saharan Africa (4). In 2019, an estimated 56 million cases of HF worldwide were recorded with a prevalence of about 712 per 100,000; this number has been in slight decline since 1990 but with some ups and downs in the years just before it (5). More recent estimates of the GBD Study 2021 suggest a reversal of this trend, with a 5.5% increase in the age-standardized prevalence from 1990 to 2021. This increase is primarily observed in regions with a high sociodemographic index (SDI) regions and is projected to continue rising until 2050 (6).

The available literature shows a complex relationship between cardiovascular conditions and mental health disorders. HF patients tend to have higher depressive symptoms and a lower quality of life (7). Recent studies also suggest an increase in suicidal thoughts and attempts among people with chronic cardiovascular conditions like HF. This highlights the psychological distress in this group (8, 9). However, the evidence is still incomplete. The methods used vary, the samples are often small, and the results are not consistently reported. This makes it difficult to draw firm conclusions.

Current research has not truly quantified the risk of suicide, nor has it established the clinical and demographic risk factors that serve to enhance suicidal behavior in this at-risk population (10). Such gaps in the literature, coupled with the importance of the increased suicide risk among HF patients, warrant a systematic review and meta-analysis to collate evidence to heighten clinical awareness and promote preventive measures.

The aim of this study is to systematically assess the risk of suicide among HF patients and to identify the demographic and clinical factors that may affect suicidal behaviors. This shall also be helpful from a screening perspective, enabling early detection and eventually targeting treatment avenues for better outcomes and improved standards of care.

## Methods

### Protocol and registration

This systematic review, together with meta-analysis, followed the Preferred Reporting Items for Systematic Reviews and Meta-Analyses (PRISMA) 2020 guidelines (11).

### Eligibility criteria

Studies needed to fulfill these requirements for inclusion in the analysis:

- The investigation targeted adult patients aged 18 and above with a confirmed heart failure diagnosis based on appropriate diagnostic criteria, regardless of disease cause or severity.
- Exposure included all forms of heart failure without restrictions, including congestive heart failure and diastolic heart failure, provided the condition met recognized diagnostic criteria.
- Studies needed to measure suicide risk (determined as ideation or planning, or attempts) using validated assessment tools, including clinical evaluations or self-report instruments.
- Only observational studies, including cohort and case-control designs, were eligible for meta-analysis. Cross-sectional studies were narratively reviewed but excluded from the pooled estimates
- Research publications needed to be either in English or the Chinese language.
- The research included peer-reviewed full-text articles.

This study did not consider reviews or book chapters or case reports or case series or letters or editorials or commentary articles, or studies that lacked suicide or self-injury outcomes or focused only on non-suicidal self-injury or self-injury without suicide risk, and did not assess ideation or planning, or attempts.

### Information sources and search strategy

A comprehensive search was conducted across several electronic databases such as PubMed/MEDLINE, Web of Science, and Scopus, from inception through June 2025. The search used a combination of Medical Subject Headings (MeSH) and relevant keywords tied to “heart failure” and “suicide, including cardiac dysfunction, heart failure, dilated cardiomyopathy, CHF, DHF, and suicide, self-harm, and self-injury. A manual hand-searching of the reference lists of included articles was conducted to identify additional eligible studies. Grey literature sources (e.g., conference proceedings, dissertations) were not included. Full details of each database’s search strategy can be found in the **Supplementary Material**.

### Study selection

All identified references were imported into EndNote, where duplicates were automatically removed. The titles and abstracts were screened to assess their relevance. All titles and abstracts were screened twice by the author to minimize errors and resolve disagreements or misinterpretations. Full texts of potentially eligible studies were then reviewed to determine final inclusion.

### Data extraction

I created and tested a standardized form to extract key information from each study. Collected data included: first author, publication year, country of study, study design and setting, sample size, participant characteristics (e.g., average age, gender distribution), heart failure classification (such as HFrEF or HFpEF, if reported), and suicide-related measures (such as type, frequency, and method of evaluation). Data was extracted and then double-checked for accuracy.

### Data synthesis and statistical analysis

A meta-analysis using a random-effects model with Restricted Maximum Likelihood (REML) estimation was performed to account for significant heterogeneity. Effect sizes were reported as odds ratios (ORs) with 95% confidence intervals (CIs) to assess the risk of suicide in patients with heart failure in comparison with the general population with no major cardiovascular condition. Heterogeneity was assessed using Cochran’s Q test, the I² statistic, and the τ² estimate. A leave-one-out sensitivity analysis was also performed to assess the robustness of the results.

To identify possible sources of variability, subgroup analyses based on two predefined factors were done: (A) the diagnostic system used for categorizing suicidal behavior (ICD-9 or ICD-10) and (B) study design (case-control vs. cohort). All analyses were completed using STATA version 17.

### Risk of bias assessment

Risk of bias of the included studies was assessed independently by two authors using the Newcastle–Ottawa Scale (NOS) criteria, which evaluates three domains including selection, comparability, and outcome (or exposure) across eight items, allowing a maximum of nine stars per study. This criterion is used for observational studies (e.g. cohorts, cross-sectional).

## Results

### Study characteristics

**Figure 1:**
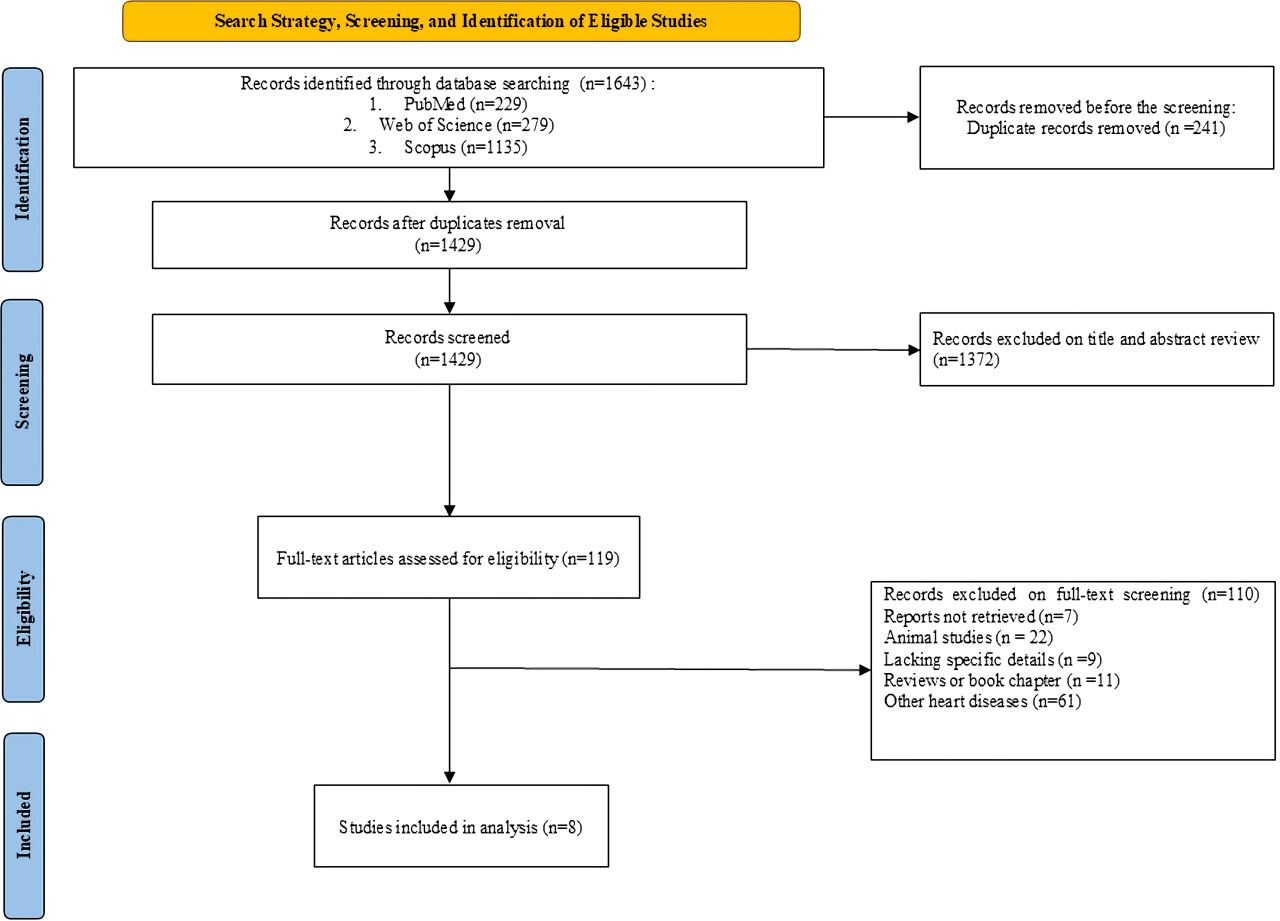
PRISMA 2020 flow diagram of the study selection process.

The study selection process is outlined in the PRISMA flow diagram (**Figure 1**). Out of the 1,643 records initially retrieved, 8 studies (12–19) met the inclusion criteria and were included in the final meta-analysis after screening and reviewing the full text.

Juurlink and colleagues conducted a case–control study in Canada, including 1,354 elderly individuals (≥66 years) who died by suicide between 1992 and 2000, with 1:4 matched living controls. Suicide deaths were identified through provincial coroners’ records, and prescription records during the preceding 6 months were reviewed. CHF was among the conditions associated with increased suicide risk (OR = 1.73; 95% CI 1.33–2.24) (12). Liu et al. conducted a case–referent study in Taiwan, including 41,050 individuals who died by suicide and 164,200 matched living controls aged ≥35 years between 2000 and 2012. Suicide deaths were ascertained from national registries. Acute coronary syndrome was significantly associated with increased odds of suicide after adjustment for psychiatric and somatic comorbidities (aOR = 1.15; 95% CI 1.05–1.26), with the highest risk during the first 6 months post-ACS (OR = 3.05; 95% CI 2.55–3.65) (13). Ahmedani et al. (14) conducted a large case–control study across eight US healthcare systems including 2,674 suicide cases and 267,400 matched controls, between 2000 and 2013. Suicide deaths were identified through healthcare records and administrative data. Seventeen physical health conditions were associated with increased suicide risk, nine of which remained significant after adjustment for mental health and substance use. Multimorbidity was present in 38% of cases versus 15.5% of controls, corresponding to nearly a two fold increased risk. Liu et al. (15) conducted a nationwide case–control study in Taiwan, including 52,749 individuals who died by suicide and 210,996 matched living controls between 2000 and 2012. Heart failure (HF) was associated with increased suicide risk (OR = 1.68; 95% CI 1.59–1.79), with the highest risk in the first 6 months after diagnosis (adjusted OR = 7.04; 95% CI 5.37–9.22). Moazzami et al. (16) analyzed 11,678 participants from NHANES 2009–2012 to examine the association between CVD and suicidal ideation (SI). SI was assessed via item 9 of the PHQ-9. Participants with CVD had a higher prevalence of SI (5.4% vs. 3.6%), with the highest in the subset with CHF and prior MI (10.6%). After adjustment for comorbidities, including depression, CVD remained significantly associated with SI (OR = 1.40; 95% CI 1.10–2.09). Crump et al. (2022) conducted a nationwide cohort study in Sweden, including 154,572 patients aged 18–75 years diagnosed with HF between 2002 and 2017 and 1,545,720 matched controls. Outcomes included new-onset major depression and suicide, ascertained via national inpatient, outpatient, and death records. HF was associated with substantially increased risks of depression and suicide, particularly in the first 3 months after diagnosis (suicide IRR in men = 4.47 [95% CI 2.62–7.62], women = 2.82 [95% CI 1.11–7.12]) (17). Stergaard et al. (18) conducted a nationwide cohort study in Denmark, including 6,635,857 individuals (2000–2020) to examine associations between 31 medical conditions and suicide. Most medical conditions (except endocrine) were associated with increased suicide risk, with a dose–response relationship observed between the disability burden of medical conditions and suicide among individuals without prior mental disorder. Yang et al. (19) conducted a community-based cohort study using UK Biobank data, including 63,923 patients first hospitalized with CVD between 1997–2020 and 127,845 matched unexposed individuals. Outcomes included psychiatric disorders and suicide attempts. CVD patients had an increased risk of psychiatric disorders and suicide attempts within the first year after hospitalization (HR = 1.83; 95% CI 1.58–2.12), largely independent of genetic susceptibility to studied psychiatric conditions. **Table 1** shows the summary of the characteristics of the included studies.

**Table 1:** Summary of characteristics of included studies.

Study	Location	Ttoal sample	Male	Female	Case	Control	Time period under observation	Source of information	Study type	Diagnostic criteria for cardiovascular diseases	Diagnostic criteria for suicide	Methods of suicide	Suicide age distribution	Non-suicide age distribution	Cardiovascular disorders
Liu, 2016 (13)	Taiwan	205,250	140,655	64595	41,050	164,200	2000-2012	NMR dataset	case-referent study	ICD-9–CM	ICD-9-CM		35–44 44696	35–44 11174	ACS, Hypertension, Dyslipidemia, CHF, Cerebrovascular disease
Juurlink 2004 (12)	America	6644	5059	1585	1329	5315	1992-2000		Case-Control Study	Consensus of 3 clinicians	clear and cogent evidence of intent in the opinion of the coroner	Hanging Firearm Self-poisoning Fall from height Drowning Stabbing	74/4	74/4	IHD+CHF+Dyslipidemia
Liu,2018 (15)	Taiwan	263,745	179955	83,790	52,749	210,996	2000-2012	NHIRD.NMR	Case-Control Study	ICD-9-CM codes 428.0 – 428.9	ICD-9-CM	not report	18–44 years: 43.36% 45–54 years: 19.89% 55–64 years: 14.04% 65–74 years: 11.22% 75 years and over: 11.48%	18–44 years: 43.36% 45–54 years: 19.89% 55–64 years: 14.04% 65–74 years: 11.22% 75 years and over: 11.48%	Congestive Heart Failure (CHF) Hypertension Ischemic Heart Disease Valve Disorders Cardiac Dysrhythmias
Ahmedani, 2017 (14)	USA	270,074	129588	140486	2674	267,400	2000-2013	VDW	Case–Control Study)	ICD 10	ICD-9	No description	49/9	39/4	Hypertension, chf,IHD,strok
Moazzami, 2018 (16)	USA	11,678	5,691	5,987	908	10,770	2009,-2010/2011-2012		case-control study	NHANES	PHQ-9	not report	66 ± 13	no	CHF CHD without MI CHD with MI
Crump, 2022 (17)	Sweden	1,700,292	99,977	54,595	154,572	1,545,720	2002-2017		Cohort Study	ICD-10	ICD-10: X60–X84	not report	18–44 years: 4.0% (140) 45–54 years: 12.1% (423) 55–64 years: 30.9% (1,079) 65–74 years: 53.0% (1,855)	18–44 years: 5.0% 45–54 years: 10.6% 55–64 years: 24.8% 65–74 years: 59.6%	HF
Yang, 2024 (19)	England	191,768	122,145	69,623	63,923	127,845	1997-2020	UK biobank	cohort	icd10	icd10	not report	63	63	Iskamics of the heart, cerebral artery disease, embolism/thrombosis, heart failure, arrhythmia
Østergaard, 2024 (18)	Denmark	6,635,857	3,298,244	3,337,613	For each case, 5 control individuals were considered.	For each case, 5 control individuals were considered.	2000-2020		Cohort	ICD 10	icd10		not report	not report	Hypertension Dyslipidemia Ischemic heart disease Atrial fibrillation Heart failure Peripheral artery occlusive disease Stroke

The PRISMA checklist for systematic reviews and meta-analyses is available in **Supplementary Material** (**Supplementary Table S2**).

### Main meta-analytic findings and influence diagnostics

A meta-analysis was carried out to aggregate findings from the selected studies and derive a comprehensive estimate of the relationship between HF and the risk of suicide. A random-effects model was employed due to anticipated clinical and methodological variations among the studies. The overall estimate indicated a significantly elevated risk of suicide in patients with HF compared to the general population (OR = 1.62, 95% CI: 1.49, 1.74). This suggests that individuals with HF have approximately 62% greater odds of committing suicide compared to control groups or the general population. Nevertheless, there was a considerable level of statistical heterogeneity (I² = 88.23%), which shows substantial variability among the results of the studies. This variability is likely due to differences in study populations, design, or diagnostic criteria utilized across the studies.

**Figure 2:**
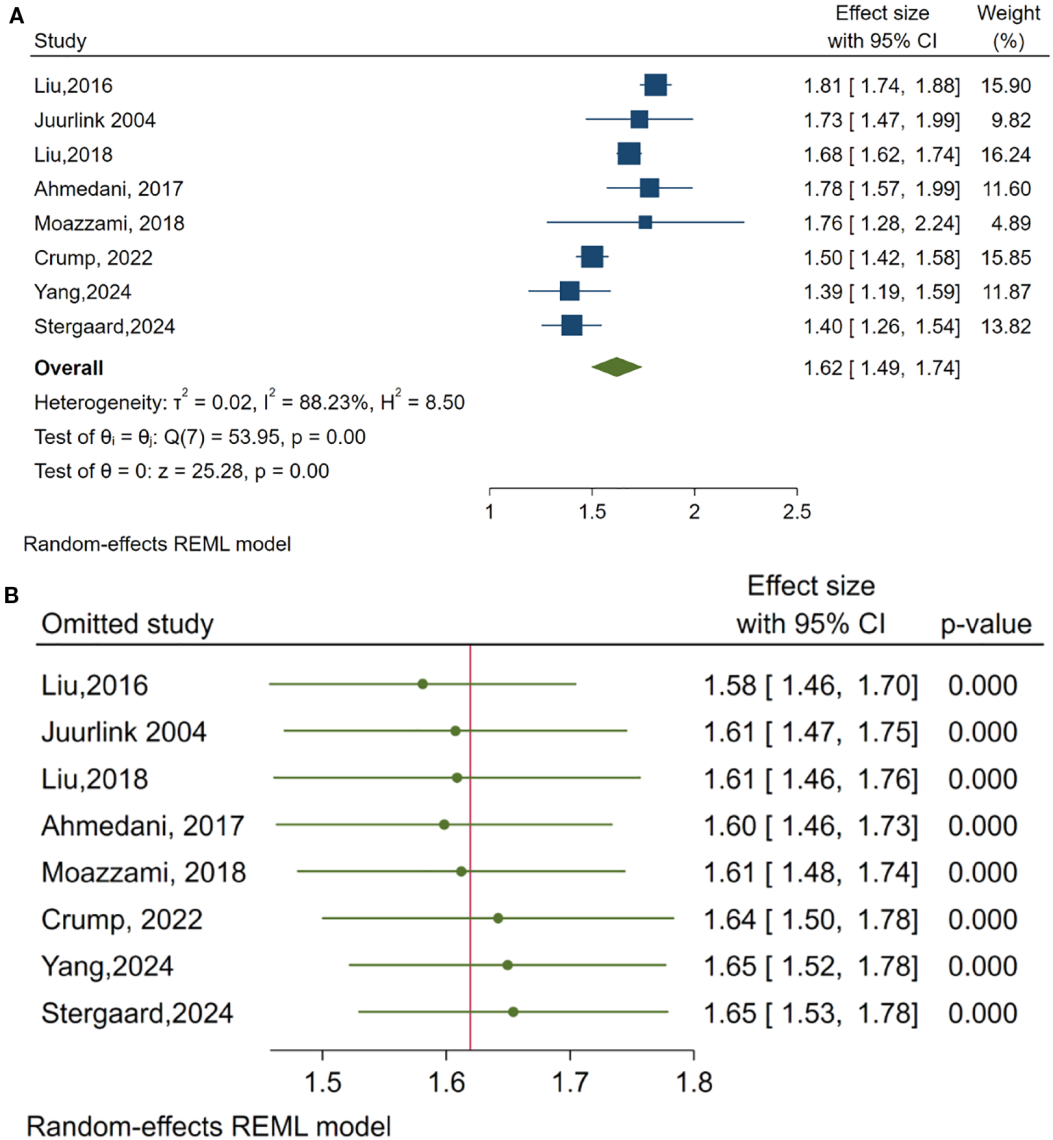
Summary of meta-analytic findings: **(A)** Forest plot of pooled effect sizes, **(B)** Leave-One-Out sensitivity analysis.

**Figure 2** depicts the principal outcomes of this meta-analysis. It effectively demonstrates both the strength and consistency of the established relationship, together with a sensitivity analysis to evaluate the robustness of the overall effect. In Panel A, the forest plot summarizes the effect sizes and their confidence intervals for individual studies, in addition to the combined overall estimate. Panel B presents a leave-one-out sensitivity analysis, indicating that no single study significantly influenced the overall result. This analysis underscores the stability and reliability of the pooled effect size, even in the presence of noted differences.

### Subgroup analyses

**Figure 3:**
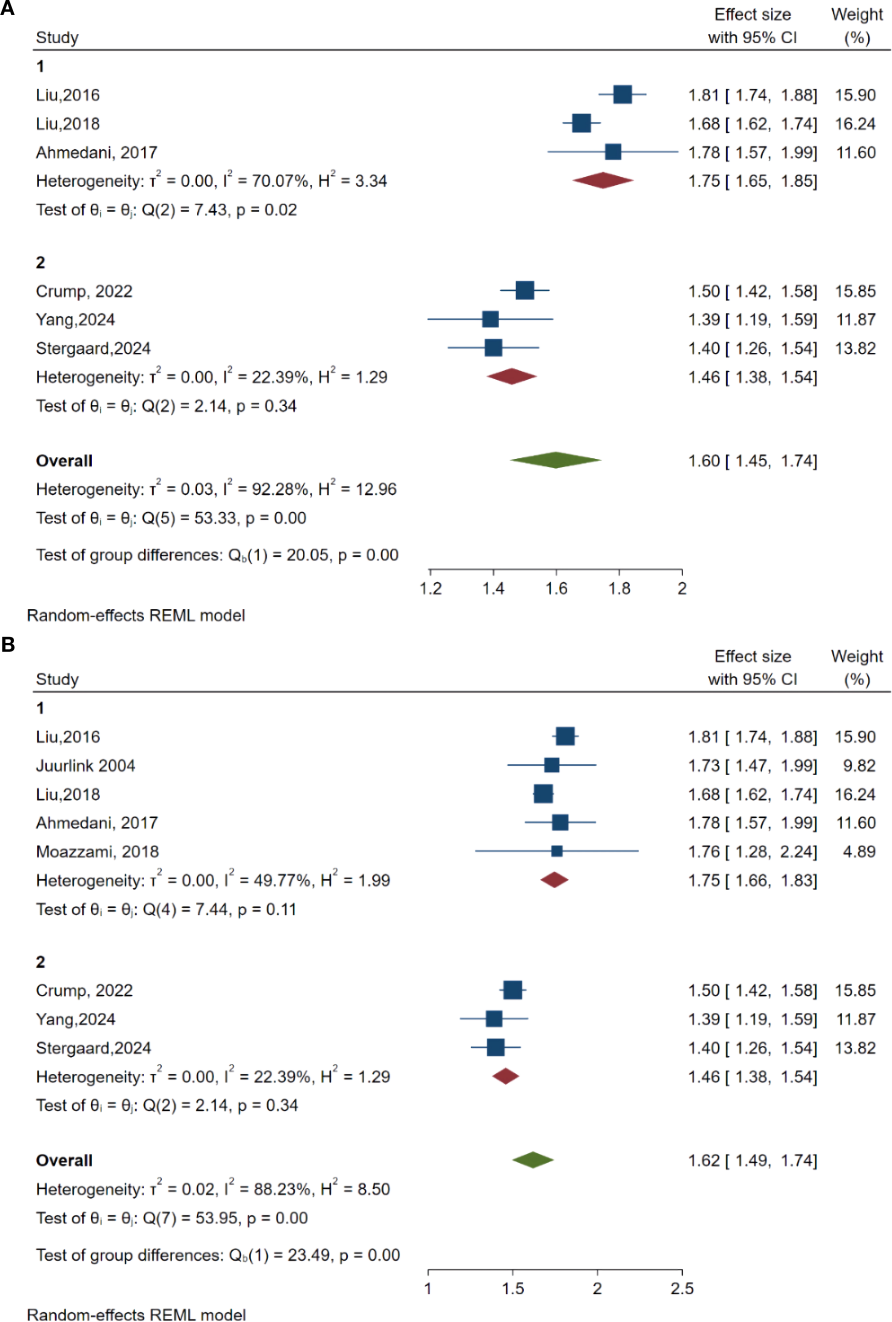
Results of subgroup analyses. **(A)** subgroup analyses based on diagnostic criteria of suicide behaviors (group 1 used ICD-9 criteria and group 2 used ICD-10 criteria). **(B)** Subgroup analyses-based study design (group 1 was case studies and group 2 was cohorts). P value 0.000 meaning the P value under p < 0.001.

To further investigate the sources of differences and identify possible effect modifiers, subgroup analyses were conducted based on two pre-established factors: (A) diagnostic criteria for suicidal behaviors (ICD-9 versus ICD-10) and (B) study design (case-control versus cohort studies) (**Figure 3**).

In Panel A, studies were categorized according to diagnostic criteria. The pooled OR for studies utilizing ICD-9 criteria was 1.75 (95% CI: 1.65, 1.85). This points to a stronger association between HF and suicide risk in comparison to studies employing ICD-10 criteria, which reported a pooled OR of 1.46 (95% CI: 1.38, 1.54). While the variation within the groups was moderate (ICD-9 group: I² = 70.07%; ICD-10 group: I² = 22.39%), the test for differences between the groups was statistically significant (Q = 20.05, p < 0.001). This implies that differences in diagnostic criteria play a significant role in the observed variations.

Panel B illustrates the subgroup analysis based on study design. The pooled OR for case-control studies was greater (OR = 1.75, 95% CI: 1.66, 1.83) than that for cohort studies (OR = 1.46, 95% CI: 1.38, 1.54). The variation within case-control studies was moderate (I² = 49.77%), while it was lower among cohort studies (I² = 22.39%). The disparity between these two groups was statistically significant (Q = 23.49, p < 0.001), highlighting study design as another significant source of variability and reinforcing the strength of findings across various study methodologies.

### Publication bias

**Figure 4:**
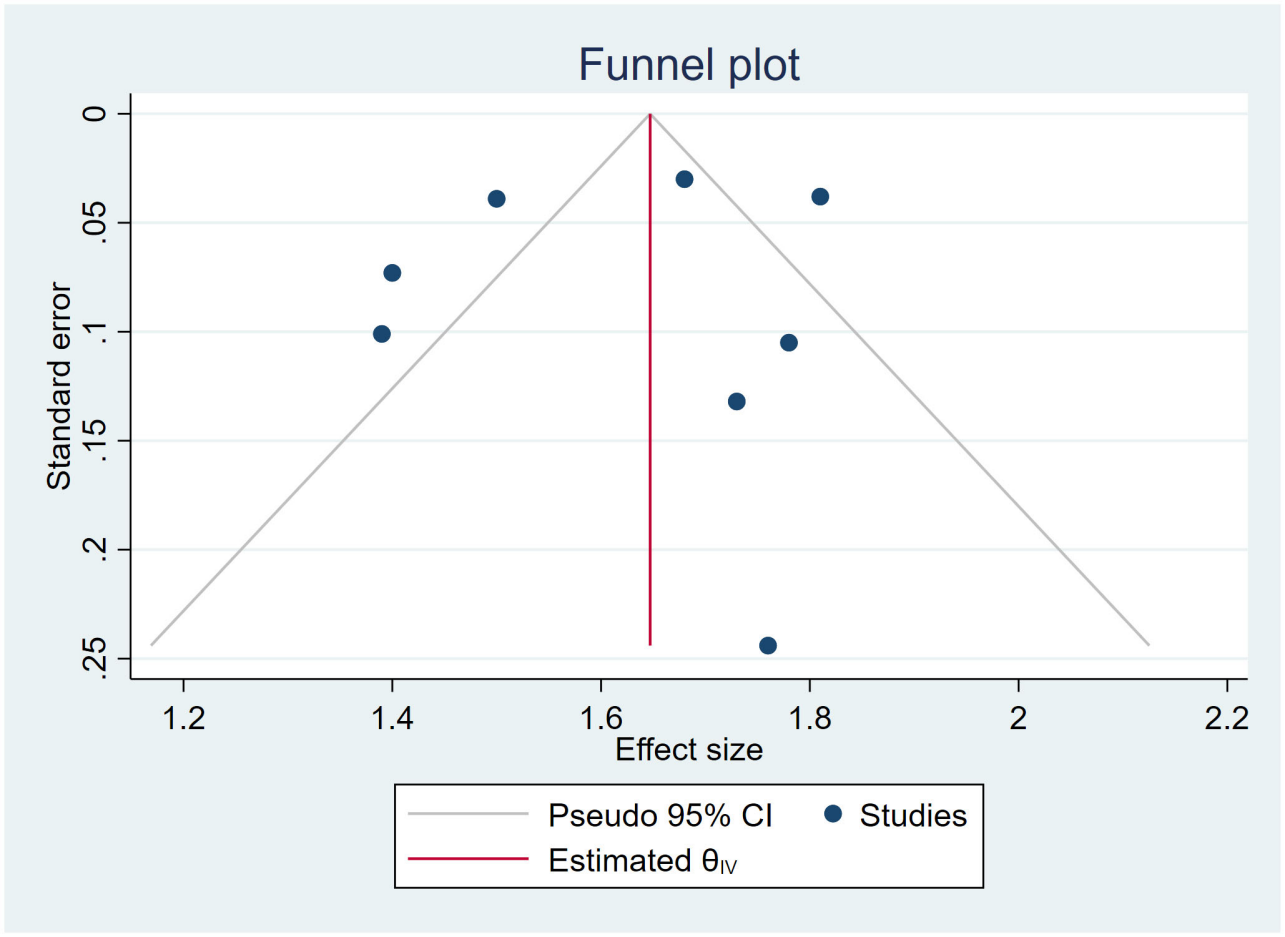
Funnel plot assessing risk of publication bias among included studies.

Visual inspection of the funnel plot (**Figure 4**) demonstrated slight asymmetry, raising the possibility of publication bias. In addition, the funnel plots show that studies with lower sample sizes or higher standard errors may not be published. Egger’s test was conducted to evaluate funnel plot asymmetry, yielding beta1 = 0.34, p = 0.793, suggesting a non-significant publication bias. However, the number of studies was limited. Thus, the observed asymmetry may be attributable to heterogeneity or chance rather than systematic bias.

### Risk of bias assessment

The score for the risk of bias assessment for each item (selection, comparability, and outcome) and total evaluation of included studies are summarized in **Table 2**. While most of the studies in our study (7 out of 8) had scores between 7 and 8, which indicates low risk of bias, one study, Moazzami (16), had a score of 6, which indicates moderate risk, mainly because of lower comparability ratings. Despite this, the study was included in the quantitative synthesis because it was proper in its methodology and relevant to the research question, and its inclusion did not significantly change the overall pooled estimates.

**Table 2:** Summary of risk of bias assessment for each included study, categorized by NOS domains.

Study	Selection	Comparability	Outcome	Total Score	Risk assessment
Liu, 2016 (13)	4	2	2	8	Low Risk
*Juurlink, 2004* (12)	4	2	1	7	Low Risk
Liu, 2016 (13)	4	2	2	8	Low risk
Ahmedani, 2017 (14)	4	2	2	8	Low Risk
Moazzami, 2018 (16)	3	1	2	6	Moderate Risk
Casey Crump, 2022 (17)	4	2	2	8	Low Risk
Jie Yang, 2024 (19)	4	2	2	8	Low Risk
Østergaard,, 2024 (18)	4	2	2	8	Low Risk

## Discussion

This review and meta-analysis studied the association between HF and the risk of suicide in adults. There was a significantly heightened risk for suicide among HF patients, whose overall OR was 1.62 (95% CI: 1.49–1.74). Sensitivity analyses reinforced the magnitude of this finding, and subgroup analyses showed variability based on the way suicidal behavior had been classified and on the study design.

HF is associated with a grave prognosis, frequently on the same level as or worse than many malignancies. Traditional epidemiological data, including the Framingham studies, indicate 20–30% in-cause mortality within the first year after diagnosis and approximately 50% mortality at five years (20). More current data verify these depressing figures. A 2007–2014 Chinese cohort study, for example, had a 17% mortality at five years after admission for HF (21). Correspondingly, international registries such as REPORT-HF, in 44 nations worldwide, have demonstrated approximately 20% mortality within the first year after discharge (22). Cardiovascular reasons dominate the most common causes of death, though non-cardiovascular reasons feature significantly in some groups. Generally speaking, these figures indicate the continuing resultant extremely high mortality from any source among HF patients, often over 10% within the first year and 50% within five years (20).

Around the globe, HF mortality rates vary unevenly despite patient demographics, comorbidities, and healthcare accessibility. The INTER-CHF study (23) was performed on 5,823 HF patients from Africa, Asia, the Middle East, and South America, and observed significant disparities in mortality within the first year: 34% in Africa, 23% in India, approximately 9% in South America and the Middle East, but just 7% in China. When the clinical variations were removed in the analysis, the findings still showed broader systemic healthcare disparities. Low-income countries had drastically poorer results. The Global Congestive HF Registry (22) proved 7.8 yearly mortality rates per 100 person-years in the high-income nations compared with 19.1 in the low-income nations. In striking contrast, European and North American research regularly shows 10-15% mortality at 1 year in the situations of well-managed and stable HF (23), reiterating the influence of geography and healthcare resources on mortality.

Psychiatric illnesses are common in HF patients and profoundly influence their overall outcomes. Depression and anxiety may act as mediators in the pathway between HF diagnosis and suicidality. Identifying and managing these symptoms is thus pivotal in suicide prevention (7). From meta-analyses, 25% to nearly 50% of HF patients are estimated to be depressed, depending on whether the criteria are suggestive of clinical depression (24.7%) or more widely depressive symptoms (41.9%) (24, 25). Anxiety also occurs in about 40% of patients, many more times than in the general population (26). Post-traumatic stress disorder (PTSD) may also occur in the aftermath of traumatic cardiac illness presentations, such as heart attacks or cardiac arrest, with 12–24% of patients at risk of developing symptoms (27). All these co-morbid psychological illnesses have dire consequences. Depression is a particularly ominous independent predictor of adverse health outcomes in the form of more hospital attendances, more healthcare costs, and more mortality. Depressed HF patients are twice as likely to reattend the emergency center or readmission back into the hospital (28).

Suicide poses a serious, though often overlooked, risk among HF patients, particularly given the high rates of depression in this group. A major Swedish study (2022) found a sharp increase in suicide risk following an HF diagnosis, especially within the first three months. The disproportionate suicide risk in men may reflect gender-specific coping mechanisms and under-recognition of depression (17). During this period, men with HF were 4.5 times more likely to die by suicide, and women were 2.8 times more likely, compared to the general population. While these suicides represent a small portion of overall deaths, the relative risk is stark and underscores the psychological burden of a life-altering HF diagnosis. Even a year after diagnosis, the suicide risk remained meaningfully elevated (17).

These findings closely follow prior research and confirm prior findings. Liu et al. (15) observed a significantly adjusted OR of 1.68 for suicide among HF patients in Taiwan, particularly in the first six months after diagnosis. Similarly, Wu et al. (29) observed a standardized mortality ratio (SMR) of 2.10 for suicide among HF patients. The disproportionate suicide risk in men may reflect gender-specific coping mechanisms and under-recognition of depression. Crump et al. (17) observed a 3- to 4-fold augmentation in the risk of suicide within the first 3 months after HF diagnosis. Petersen et al. (30) observed a corresponding increase in immediate risk at diagnosis in a cohort from Denmark. On smaller scales, Korkmaz et al. (31) and Shofu-Akanji et al. (32) provided additional confirmation by assigning heightened ideas on committing suicide to the psychosocial phenomena of hopelessness and low self-esteem. A systematic review by Chi et al. (33) observed a pooled OR of about 1.7 and validated the present findings.

In addition, depression elevates mortality in the broad population of HF patients. Major depression patients are 1.5 to 2 times at risk of dying compared with non-depressed patients. Suicidal ideation by itself similarly foretells elevated mortality. A United States Veterans Affairs study illustrates the former by demonstrating that elderly HF patients admitted to nursing homes with suicidal ideation experienced more than twice the 30-day mortality, even after the multivariable adjustment for other medical or psychiatric illnesses (34).

Clinically, the heightened risk for suicide in HF patients mandates extensive mental health screening in the initial phase following diagnosis or hospitalization. Widespread utilization of brief instruments to gauge suicidal ideation may enable prompt intervention. Care models, including the incorporation of the fields of cardiology, psychiatry, and social work may potentially reduce the risk. Policymakers need to take into consideration the inclusion in HF management guidelines of mental health screenings (35).

This analysis identifies numerous strengths and weaknesses. With the help of subgroup analyses and sensitivity tests, the analysis identified sources of heterogeneity and the reliability of our results. The large, aggregated sample size, the implementation of REML modeling, and the inclusion of current studies from diverse fields make our results more credible and transferable. A Major limitation is the lack of detailed reporting on HF subtypes (e.g., HFrEF, HFpEF, acute vs. chronic) and the severity of psychiatric comorbidities. Additionally, definitions of suicide varied across studies, which may have influenced outcome classification. Data on pharmacological or psychotherapeutic interventions such as antidepressant use or counseling were largely absent, limiting our ability to explore their potential modifying effect on suicide risk and to draw firm causal inferences. Furthermore, the observational design of the included studies precludes establishing causal relationships. Many studies did not adequately control for relevant psychiatric comorbidities, such as depression, anxiety disorders, or substance abuse, nor for antidepressant drug use, all of which are recognized modifiers of suicide risk; this may have introduced residual confounding that affected the observed associations. Important psychosocial factors and detailed measures of heart failure severity were often missing. The relatively small number of eligible studies also limited our ability to conduct a meta-regression based on variables like location sample size, race, etc., which could be a source of heterogeneity among included studies. Finally, heterogeneity in study design, follow-up duration, and outcome definitions may further restrict the comparability and generalizability of findings. Future research should focus on prospective cohort analyses performed through standard instruments for the assessment of suicide risk and comprehensive clinical evaluation. To better understand and prevent suicidal risk in heart failure patients, future research should assume an interdisciplinary approach that integrates perspectives from cardiology, psychiatry, psychology, and public health. Multidisciplinary, prospectively designed studies must be conducted on a large scale to capture psychosocial factors, such as social support, quality of life, and history of mental illness, as well as clinical information. Novel, validated, comprehensive risk stratification instruments that incorporate biomedical, psychiatric, and social determinants shall be paramount for identifying high-risk individuals and instituting appropriate, timely interventions.

## Conclusion

Overall, the current systematic review and meta-analysis confirm a clear link between HF and the risk for suicide. The findings show suicide risk in HF patients as being significant and changing over time. Risk is heightened in the immediate phase at diagnosis and in patients with severe or poorly controlled disease. Such findings stress the requirement for active mental health assessment, screening, and focused interventional strategies in the therapeutic protocols for HF patients.

## Data Availability

The original contributions presented in the study are included in the article/**Supplementary Material**. Further inquiries can be directed to the corresponding author.
