# Supplementary material for: Assessing suicide risk in patients with heart failure: a systematic review and meta-analysis
Source: Front Psychiatry. 2025 Sep 17;16:1674302. doi: 10.3389/fpsyt.2025.1674302 (PMC12484135; doi:10.3389/fpsyt.2025.1674302)
Supplement: Supplementary file 2 [file Table2.docx]

| Study | Location | Ttoal Sample | Male | Female | Case | Control | Time Period Under Observation | Source of Information | Study type | Diagnostic Criteria for Cardiovascular Diseases | Diagnostic Criteria for Suicide | Methods of suicide | Suicide Age distribution | Non-Suicide Age distribution | Cardiovascular disorders |
| --- | --- | --- | --- | --- | --- | --- | --- | --- | --- | --- | --- | --- | --- | --- | --- |
| Liu,2016 | Taiwan | 205,250 | 140,655 | 64595 | 41,050 | 164,200 | 2000-2012 | NMR dateset | case-referent study | ICD-9–CM | ICD-9-CM |  | 35–44 44696 | 35–44 11174 | ACS, Hypertension, Dyslipidemia, CHF, Cerebrovascular disease |
| Juurlink 2004 | Canada | 6644 | 5059 | 1585 | 1329 | 5315 | 1992-2000 |  | Case-Control Study | Consensus of 3 clinicians | clear and cogent evidence of intent in the opinion of the coroner | Hanging Firearm Self-poisoning Fall from height  Drowning Stabbing | 74/4 | 74/4 | IHD+CHF+Dyslipidemia |
| Liu,2018 | Taiwan | 263,745 | 179955 | 83,790 | 52,749 | 210,996 | 20,002,012 | NHIRD.NMR | Case-Control Study | ICD-9-CM codes 428.0 – 428.9 | ICD-9-CM | not report | 18–44 years: 43.36%  45–54 years: 19.89%  55–64 years: 14.04%  65–74 years: 11.22%  75 years and over: 11.48% | 18–44 years: 43.36%  45–54 years: 19.89%  55–64 years: 14.04%  65–74 years: 11.22%  75 years and over: 11.48% | Congestive Heart Failure (CHF) Hypertension  Ischemic Heart Disease  Valve Disorders  Cardiac Dysrhythmias |
| Ahmedani, 2017 | USA | 270,074 | 129588 | 140486 | 2674 | 267,400 | 2000-2013 | VDW | Case–Control Study) | ICD 10 | ICD-9 | No description | 49/9 | 39/4 | Hypertension, chf,IHD,strok |
| Moazzami, 2018 | USA | 11,678 | 5,691 | 5,987 | 908 | 10,770 | 2009,-2010/2011-2012 |  | case-control study | NHANES | PHQ-9 | not report | 66 ± 13 | no | CHF CHD without MI CHD with MI |
| Crump, 2022 | Sweden | 1,700,292 | 99,977 | 54,595 | 154,572 | 1,545,720 | 2002-2017 |  | Cohort Study | ICD-10 | ICD-10: X60–X84 | not report | 18–44 years: 4.0% (140 ) 45–54 years: 12.1% (423 ) 55–64 years: 30.9% (1,079 ) 65–74 years: 53.0% (1,855 ) | 18–44 years: 5.0% 45–54 years: 10.6% 55–64 years: 24.8% 65–74 years: 59.6% | HF |
| Yang,2024 | England | 191,768 | 122,145 | 69,623 | 63,923 | 127,845 | 1997-2020 | UK biobank | cohort | icd10 | icd10 | not report | 63 | 63 | Iskamics of the heart, cerebral artery disease, embolism/thrombosis, heart failure, arrhythmia |
| Stergaard,2024 | Denmark | 6,635,857 | 3,298,244 | 3,337,613 | For each case, 5 control individuals were considered. | For each case, 5 control individuals were considered. | 2000-2020 |  | Cohort | ICD 10 | icd10 |  | not report | not report | Hypertension Dyslipidemia Ischemic heart disease Atrial fibrillation Heart failure Peripheral artery occlusive disease Stroke |
