# Supplementary material for: Assessing suicide risk in patients with heart failure: a systematic review and meta-analysis
Source: Front Psychiatry. 2025 Sep 17;16:1674302. doi: 10.3389/fpsyt.2025.1674302 (PMC12484135; doi:10.3389/fpsyt.2025.1674302)
Supplement: Supplementary file 3 [file Table3.docx]

| **Study** | **Selection** | **Comparability** | **Outcome** | **Total Score** | **Risk assessment** |
| --- | --- | --- | --- | --- | --- |
| **Liu,2016** | 4 | 2 | 2 | 8 | Low Risk |
| ***Juurlink, 2004*** | 4 | 2 | 1 | 7 | Low Risk |
| **Liu ,2016** | 4 | 2 | 2 | 8 | Low risk |
| **Ahmedani, 2017** | 4 | 2 | 2 | 8 | Low Risk |
| **Moazzami,2016** | 3 | 1 | 2 | 6 | Moderate Risk |
| **Casey Crump,2022** | 4 | 2 | 2 | 8 | Low Risk |
| **Jie Yang,2024** | 4 | 2 | 2 | 8 | Low Risk |
| **stergaard,2024** | 4 | 2 | 2 | 8 | Low Risk |
